# Development of Highly Sensitive Digital Droplet PCR for Detection of cKIT Mutations in Circulating Free DNA That Mediate Resistance to TKI Treatment for Gastrointestinal Stromal Tumor (GIST)

**DOI:** 10.3390/ijms24065411

**Published:** 2023-03-12

**Authors:** Michael Rassner, Silvia Waldeck, Marie Follo, Stefanie Jilg, Ulrike Philipp, Martina Jolic, Julius Wehrle, Philipp J. Jost, Christian Peschel, Anna Lena Illert, Justus Duyster, Florian Scherer, Nikolas von Bubnoff

**Affiliations:** 1 Department of Medicine I, Medical Center—University of Freiburg, Faculty of Medicine, University of Freiburg, 79085 Freiburg, Germany; 2 German Cancer Consortium (DKTK) Partner Site Freiburg and German Cancer Research Center (DKFZ), 69120 Heidelberg, Germany; 3 III Medical Department for Hematology and Oncology, Klinikum Rechts der Isar, Technische Universität München, 80333 Munich, Germany; 4 Onkologie Erding, 85435 Erding, Germany; 5 Department of Biomaterials, Sahlgrenska Academy, University of Gothenburg, 405 30 Gothenburg, Sweden; 6 Department of Clinical Oncology, Division of Internal Medicine, Medical University of Graz, 8036 Graz, Austria; 7 Department of Hematology and Oncology, Medical Center, University of Schleswig Holstein, Campus Lübeck, Ratzeburger Allee 160, 23538 Lübeck, Germany

**Keywords:** ddPCR, GIST, cKIT, PDGFRA, ctDNA, liquid biopsy, biomarkers

## Abstract

Background: Mutations in cKIT or PDGFRA are found in up to 90% of patients with gastrointestinal stromal tumors (GISTs). Previously, we described the design, validation, and clinical performance of a digital droplet (dd)PCR assay panel for the detection of imatinib-sensitive cKIT and PDFGRA mutations in circulating tumor (ct)DNA. In this study, we developed and validated a set of ddPCR assays for the detection of cKIT mutations mediating resistance to cKIT kinase inhibitors in ctDNA. In addition, we cross-validated these assays using next generation sequencing (NGS). Methods: We designed and validated five new ddPCR assays to cover the most frequent cKIT mutations mediating imatinib resistance in GISTs. For the most abundant imatinib-resistance-mediating mutations in exon 17, a drop-off, probe-based assay was designed. Dilution series (of decreasing mutant (MUT) allele frequency spiked into wildtype DNA) were conducted to determine the limit of detection (LoD). Empty controls, single wildtype controls, and samples from healthy individuals were tested to assess specificity and limit of blank (LoB). For clinical validation, we measured cKIT mutations in three patients and validated results using NGS. Results: Technical validation demonstrated good analytical sensitivity, with a LoD ranging between 0.006% and 0.16% and a LoB ranging from 2.5 to 6.7 MUT fragments/mL. When the ddPCR assays were applied to three patients, the abundance of ctDNA in serial plasma samples reflected the individual disease course, detected disease activity, and indicated resistance mutations before imaging indicated progression. Digital droplet PCR showed good correlation to NGS for individual mutations, with a higher sensitivity of detection. Conclusions: This set of ddPCR assays, together with our previous set of cKIT and PDGFRA mutations assays, allows for dynamic monitoring of cKIT and PDGFRA mutations during treatment. Together with NGS, the GIST ddPCR panel will complement imaging of GISTs for early response evaluation and early detection of relapse, and thus it might facilitate personalized decision-making.

## 1. Introduction

Oncogenic mutations in the stem cell factor receptor tyrosine kinase (cKIT) or the platelet-derived growth factor receptor alpha (PDGFRA) are found in 85–90% of patients with gastrointestinal stromal tumors (GISTs) [1]. cKIT mutations in GISTs comprise substitutions, various deletions, insertions, deletion-insertions, and duplications [2,3,4]. The most common mutations are located in exon 11 (~70% of cases [5]), affecting the negative-regulatory, intracellular, juxtamembrane domain and causing constitutive receptor activation [6]. cKIT exon 11 deletions are the most frequent aberrations in GISTs, followed by exon 11 substitutions [3]. Mutations of exon 9, which encodes the extracellular domain, are found in 10–15% of cases and mostly constitute the A502-Y503dup [5]. Variants affecting cKIT exon 13 are rare and mostly emerge as K642E exchange [3,7]. In 10–15% of GIST cases, PDGFRA substitutions can be found, mostly comprising an activating exon 18 D842V exchange, which encodes the activation domain that causes primary imatinib resistance [8]. Deletions in exon 18 are less common [3,7,8]. Mutations emerging with treatment are associated with imatinib resistance, are less heterogeneous than primary mutations, and occur in two regions: the ATP binding pocket encoded by cKIT exon 13/14 or the activation loop encoded by exon 17/18 [7,9]. These mutations are predominantly point mutations that lead to amino acid exchanges, e.g., exon 13 V654A, exon 14 T670I, or exon 17 D820Y, N822K, or Y823D [7,9].

Genotyping of GISTs is mandatory for all patients before treatment. The genotype predicts the response to treatment and is used for treatment stratification [8]. In addition, specific mutations in cKIT/PDFGRA interfere with drug binding and are associated with treatment resistance and disease progression [2,6].

In GISTs, treatment response is assessed by imaging [10]. However, this method displays limited sensitivity and specificity [10]. Currently, there are no other biomarkers available for treatment monitoring. In addition, repeated genotyping requires repetitive biopsies that are invasive and, as a consequence of sampling bias, do not represent the clonal composition of the disease [11]. Clonal evolution is of clinical relevance, since subclones harboring mutations that mediate drug resistance will be selected in patients receiving cKIT-targeted drugs [12], and detection of the predominant mutation may direct the next line of therapy [13,14]. 

Profiling of circulating tumor DNA (ctDNA) has emerged as a powerful tool to genotype and monitor malignant tumors noninvasively from blood plasma [15,16]. The amount and composition of ctDNA have been shown to mirror disease activity and clonal evolution in NSCLC, breast cancer, colorectal cancer, and melanoma [6,17,18,19]. As an example, in EGFR-mutated metastatic NSCLC, monitoring of EGFR T790M in ctDNA can be used to predict the response to the T790M-specific inhibitor osimertinib [19,20,21].

Techniques for ctDNA detection can be divided into targeted, single-mutation approaches (PCR: qPCR, BEAMing, ddPCR) or more unbiased approaches by means of targeted next-generation sequencing (NGS) [9,22]. NGS is more advantageous than single-mutation assays, as it allows for simultaneous genome-wide testing in a single assay [23] and it was shown to mirror ctDNA clonal heterogeneity in GIST patients [11,24,25,26,27,28,29]. On the other hand, NGS-based approaches are expensive and time-consuming and still demand an extensive bioinformatic analysis [30,31,32,33]. Digital droplet PCR emerged as a robust, easy-to-use, and cost-effective method with high reproducibility, without the need for bioinformatics [31]. It is conceptually simple and a powerful method for accurate quantification of a minimal amount of ctDNA without preamplification [22,31,34]. 

We have previously shown that the combination of ligation PCR (L-PCR) and ddPCR detected ctDNA in 92% of 25 GIST patients with active disease, with ddPCR displaying distinctly improved sensitivity and specificity [30]. In this study, a targeted NGS panel detected additional driver mutations, including exchanges mediating imatinib resistance.

In the current study, we developed and validated a set of GIST-specific ddPCR assays for the detection of the most frequent cKIT mutations mediating imatinib resistance. The performance of these assays was clinically validated in three exemplary patients and technically validated using NGS.

## 2. Results

**Figure 1:**
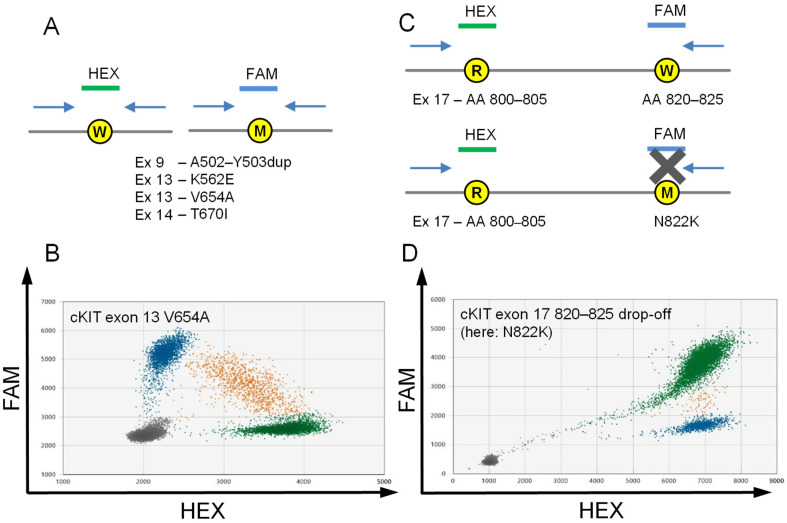
Principle of a wildtype (WT)/mutation probe assay and the „drop-off“ assay. (**A**,**B**) For the substitution mutations (here exemplary cKIT exon 13 V654A), a HEX-labeled probe binds to the WT strand (green) and a FAM-labeled probe binds to the mutant strand (blue). Double positive droplets (orange) contain both WT and mutant DNA. (**C**,**D**) In case of heterogenous cKIT exon 17 (here exemplary N822K), a HEX-labeled reference probe binds to both the WT and mutated strands, while the FAM-labeled hotspot drop-off probe only binds to the WT, thus resulting in a double-positive signal for the WT ((**D**), green) and a single-positive signal for the mutant ((**D**), blue). A small number of droplets lies in between (orange), corresponding to WT- and mutant-DNA-containing droplets, which show less FAM signal.

We designed four ddPCR assays to cover the most frequent cKIT mutations mediating drug resistance to cKIT–ATP competitor-type kinase inhibitors in GISTs (Figure 1; Table 1, Supplementary Materials Table S1). 

Furthermore, we redesigned our previous cKIT exon 9 A502-Y503dup assay, switching from a drop-off based [30] to a WT/MUT probe-based assay for more convenient readout. We also designed an assay for the most frequent primary cKIT exon 13 mutation K642E (Figure 1A,B). All assays were designed and validated with recombinant gene fragments and customized primers and probes. The components were then tested in temperature gradients, under adjusted annealing times, cycling numbers, and primer concentrations.

### 2.1. Partition Number, Mean Copies per Partition, Individual Partition Volume, and Total Partition Volume

The partition number, mean copies per partition, and total volume of the partitions (effective reaction size) are given in Table 1. The individual partition volume was approximately 1 nL. For assay testing and validation, a defined copy number per partition was applied.

### 2.2. Assays to Detect Stereotypic cKIT Imatinib Resistance Mutations Exon 13 V654A and Exon 14 T670I

We designed single-target ddPCR assays for the two prevailing cKIT imatinib resistance mutations exon 13 V654A and exon 14 T670I. Within these assays, a HEX-labeled LNA probe binds to the WT sequence while a FAM-labeled LNA probe is complementary to the MUT sequence (Figure 1A,B). The binding of each probe to the respective DNA sequence produces a single color signal, while droplets containing both WT and MUT DNA result in double-positive signals (Figure 1A,B). 

### 2.3. Drop-off Based Detection of cKIT Exon 17 Mutations

For the prevailing cKIT exon 17 hotspot mutations, a reference probe and a deletion hotspot drop-off probe were designed (Figure 1C,D). In this approach, the reference probe binds to an upstream sequence, present in both the WT and the MUT sequence. Mutations in cKIT exon 17 that mediate imatinib resistance cluster in D820-Y823 [2,35,36]. Accordingly, in the present study, we designed an upstream HEX-labeled reference probe that binds to D800-V805 and a FAM-labeled D820-V825 drop-off probe (Figure 1C). A WT strand produces a double-positive signal, whereas a mutation in D820-V825 results in a HEX single-positive signal.

### 2.4. Assay Validation

**Figure 2:**
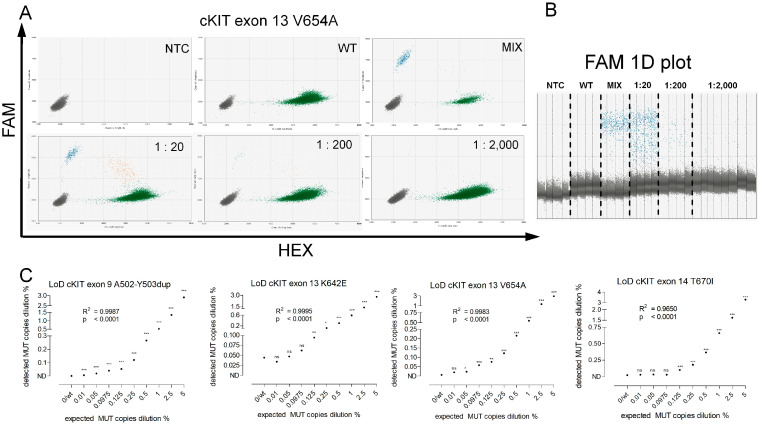
Dilution series made to determine the test sensitivity/Limit of Detection (LoD). (**A**) Decreasing amounts of mutant genomic DNA were added to a fixed copy number of wildtype (WT) human DNA to determine the assay’s ability to reliably detect the target of interest (here shown for cKIT exon 13 V654A). No-template-control (NTC) and WT-DNA-containing wells were run in parallel to exclude unspecific, i.e., false positive binding. (**B**) Dilution series in the 1D plot (depicting only the FAM = mutant signal). (**C**) Determination of the LoD for cKIT exon 9 A502-Y503dup, cKIT exon 13 K642E and V654A, and cKIT exon 14 T670I. Asterisks indicate the dilution where a two-folded *t*-test compared to NTC or WT resulted in a significant value (* *p* < 0.05, ** *p* < 0.01, *** *p* < 0.001). The minimal significant detected ratio was defined as LoD. R^2^ indicates the regression coefficient yielded by regression analysis.

By spiking in decreasing amounts of recombinant MUT DNA in a constant background of human genomic WT DNA, we determined the LoD. Dilution series were conducted for all five mutations (Table 1; exemplary panel for cKIT exon 13 V654A in Figure 2A–C). Regression analysis showed a high correlation between the expected and detected ratios (Figure 2C). The median LoD for all assays was 1:4869 (0.02%; range 0.005–0.16%). The assays provided high sensitivity. The novel WT/MUT probe-based assay for cKIT exon 9 A502-Y503dup displayed a LoD below 1:10,000 (0.01%) (Table 1). The drop-off assay for cKIT exon 17 mutations yielded a LoD of 1:610 (0.16%). All other assays resulted in LoDs of at least 1:1000 (0.1%) (Table 1).

**Figure 3:**
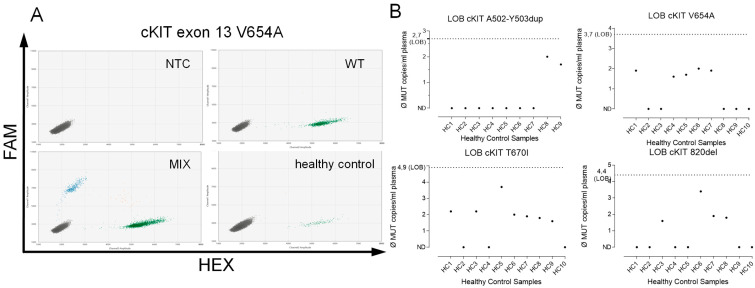
Measurements of plasma from healthy controls, taken to determine the Limit of Blank (LoB). (**A**) cfDNA isolated from plasma specimens from healthy subjects was measured to determine the LoB. The example shows cKIT exon 13 V654A (green and blue dots indicate WT or mutant fragment signal, respectively). Thus, a threshold to distinguish true positive droplets from unspecific or background (i.e., false positive) signals was determined. (**B**) Determination of the LoB as shown for cKIT exon 9 A502-Y503dup, cKIT exon 13 K642E and V654A, and cKIT exon 14 T670I. The LoB (dotted line) for each assay was calculated as the sum of the mean of all measured values plus three times the standard deviation.

To determine the LoB, cfDNA from ten healthy controls was introduced to each assay (Figure 3A,B). Thus, we determined a threshold to distinguish positive from negative partitions (Table 1). The calculated median LoB was 3.70 MUT fragments/mL (range 2.5–6.7 fragments/mL; Table 1).

### 2.5. Patient Cases

We measured blood samples from three patients with GISTs, known (from tissue sequencing and active disease) to have activating cKIT mutations, for ctDNA. The cfDNA content after isolation from plasma was determined for all samples and amounted to a median concentration of 0.9 ng/µL (range: 0.5–11.3 ng/µL).

#### 2.5.1. Case 1

**Figure 4:**
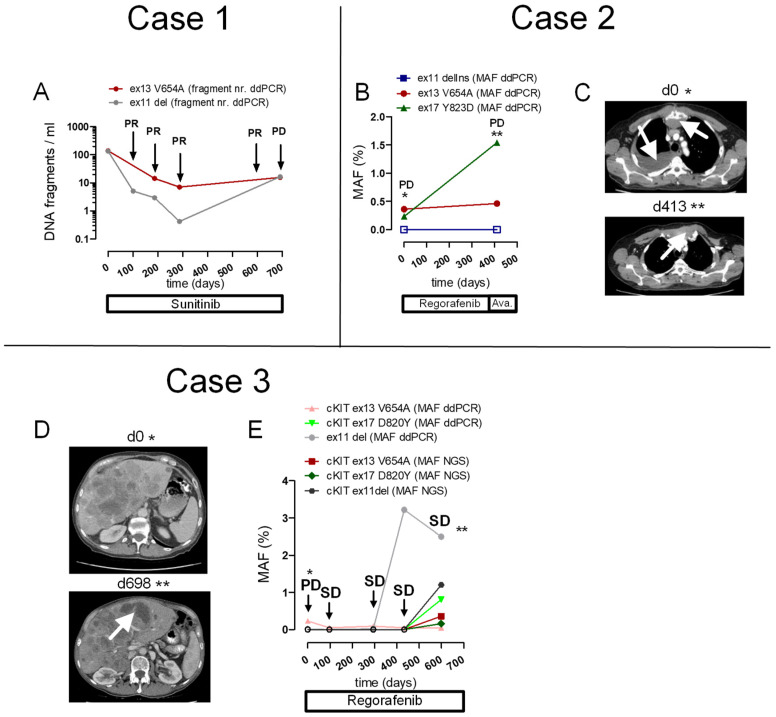
Clinical assay validation. (**A**) Results of digital droplet PCR depicting ctDNA levels over time for cKIT exon 13 V654A. The patient had a primary gastric GIST with a cKIT exon 11 Y553-Q556del identified in a tissue sample at baseline ([30]; grey). The patient was switched from adjuvant imatinib to sunitinib for peritoneal metastases. At this time, NGS revealed an imatinib-insensitive cKIT exon 13 V654A mutation. Absolute ctDNA fragment numbers of the V654A mutation declined (red) and the patient reached a partial response (PR). Eventually, ctDNA fragment numbers increased with progression. The mutant allelic fraction (MAF) measured by ddPCR reflected the course of ctDNA previously measured by NGS [30] (see Supplementary Materials Table S3). (**B**) Tracking of multiple mutations using ddPCR in a patient with duodenal GIST-positive cKIT exon 11 deletion. The patient received regorafenib because of progression of hepatic, osseous, and soft tissue metastatic lesions after previous therapy with imatinib followed by sunitinib. A cKIT 13 V654A mutation was detected from sequencing of liver tissue at the time of progression. The patient experienced further progression of a retrosternal mass while receiving regorafenib (*) and a progressive presternal mass while receiving the subsequent treatment with avapritinib (**). At this time, a liver specimen revealed a cKIT exon 17 Y823D mutation. Digital droplet PCR of cfDNA isolated from plasma samples taken one and two years before the cKIT exon 17 mutation was known from the liver specimen already showed detectable levels of this mutation at an earlier time point and an increasing MAF (green) during disease progression. Empty squares indicate negative measurements. (**C**) CT imaging of the retrosternal mass and pleural effusion at d0 (* in B, upper panel, arrows) and progression of a presternal thoracic wall mass at d413 (** in B9, lower panel, arrow). (**D**) A patient with a rectal GIST and a cKIT exon 11 deletion (W557-K558del) received multiple lines of treatment (imatinib, sunitinib, nilotinib) before treatment with regorafenib was instituted for progressive liver metastases (PD, *). Progressive hepatic lesions were observed at d0 (*, upper panel). At d698 (**) the liver metastases were again progressive, as shown exemplarily by a hypodense lesion in liver segment III/IV (lower panel, arrow). (**E**) The patient remained in stable disease (SD) for almost two years (arrows), before imaging indicated progression of the liver metastases (d698 **). Digital droplet PCR showed ctDNA positive for the cKIT exon 11 deletion with gradually increasing MAF, as assessed previously ([30]; grey). Digital droplet PCR detected ctDNA for V654A at all time points (pink triangles) whereas in matched samples NGS did not detect cKIT V654A at 4/5 time points (red squares). For cKIT D820Y, both NGS and ddPCR showed concordant results, with detection only at d599. At this time point, fragment number and MAF increased, with a higher MAF measured by ddPCR (green) than by NGS (blue). Similar to patient #1, NGS analysis resulted in lower overall MAFs compared to ddPCR. Empty circles indicate negative measurements.

This patient had a primary gastric GIST with a cKIT exon 11 Y553-Q556del known from tissue analysis at baseline. The patient received adjuvant imatinib after surgery and eventually relapsed with peritoneal metastases. Treatment was then switched to sunitinib (Figure 4A). In our previous study, we showed that following the initiation of sunitinib, the levels and MAF of the cKIT exon 11 rapidly declined and the patient achieved a partial response (patient #6 in [1]; grey in Figure 4A). Analysis of ctDNA by NGS previously indicated a cKIT exon 13 V654A mutation in ctDNA that confers imatinib resistance [30]. 

For the present study, we cross-validated these results by running ddPCR using a V654A-specific ddPCR assay and followed the course of ctDNA harboring this exchange during treatment. The course of absolute levels of ctDNA fragments and the MAF of the V654A mutation assessed by ddPCR (red in Figure 4A) corresponded to the courses of both the primary cKIT mutation (ddPCR) and the MAFs of NGS for both mutations that were previously reported [30]. Of note, ddPCR displayed a higher MAF compared to NGS except for baseline V654A, and NGS failed to detect ctDNA harboring cKIT Y553-Q556del at two out of three time points, or V654A at one out of three time points. In contrast, ddPCR detected both variants in plasma from all three time points (Supplementary Materials Table S3). Eventually, the patient experienced peritoneal progression and both the exon 11 deletion (absolute fragment number and MAF [30]) and the absolute fragment number for exon 13 cKIT mutations increased (Figure 4A).

#### 2.5.2. Case 2

This patient had a duodenal GIST with initial hepatic metastases. Tissue analysis revealed a cKIT exon 11 deletion (V559delinsN). After imatinib treatment, the patient received sunitinib because of progressive liver metastases. After 30 months of sunitinib treatment, the patient experienced progressive liver, osseous, and mediastinal metastases (≙d0 in Figure 4B,C). A hemihepatectomy was conducted, and Sanger sequencing of tumor tissue demonstrated the previously known cKIT exon 11 deletion, a novel cKIT exon 13 V654A mutation, and WT sequences for cKIT exon 9 and 17. Therapy with regorafenib was started. At day 413, the patient was switched to avapritinib because of mediastinal and thoracic wall progression (Figure 4C, lower panel, arrow). Eight months later, the patient experienced further progression of osseous and liver metastases. A rechallenge to sunitinib was initiated. However, metastatic liver lesions further progressed and Sanger sequencing from a liver biopsy yielded previously known mutations in cKIT exon 11 and 13 in addition to a cKIT exon 17 Y823D mutation. The University Medical Center Freiburg Molecular Tumor Board recommended therapy with ripretinib. However, the patient did not receive further therapy and succumbed to the progressive, metastatic GIST. Importantly, in addition to the V559delinsN and V654A exchanges, ddPCR detected the cKIT exon 17 Y823D mutation in cfDNA isolated from plasma samples taken one and two years before this mutation was detected in the liver specimen, with increasing fragment numbers and MAF (Figure 4B).

#### 2.5.3. Case 3

In our previous study, we showed that this GIST patient (#25 in [30]) developed increasing fragment numbers and MAF for a cKIT exon 11 deletion, preceding radiological progression after nine months of regorafenib treatment (Figure 4D, lower panel, white arrow and Supplementary Materials Table S3). NGS of cfDNA performed at two time points (d294 and d599) demonstrated two additional cKIT mutations, exon 13 V654A and exon 17 D820Y [30]. To validate ddPCR assays designed for V654A and D820Y, we examined samples from five time points for V654A and D820Y using ddPCR and compared results to matched samples measured by NGS (Supplementary Materials Table S3, Figure 4E). Digital droplet PCR detected ctDNA for V654A at all time points (pink triangle in Figure 4E), whereas NGS produced negative values at 4/5 time points (Supplementary Materials Table S3, red square in Figure 4E). For cKIT D820Y, both NGS and ddPCR showed concordant results, with negative values until d599, when fragment number and MAF increased. A higher MAF was observed using ddPCR (green in Figure 4E and Supplementary Materials Table S3).

### 2.6. Correlation of MAF for cKIT Mutations, Determined with Targeted NGS Versus ddPCR

**Figure 5:**
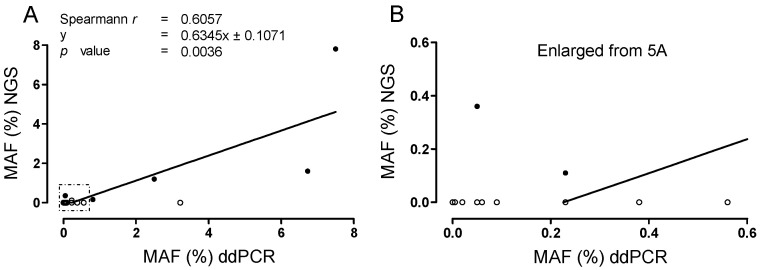
Correlation of MAF assessed with ddPCR versus NGS. (**A**) For test validation, ctDNA for cKIT exon 11, exon 13 V654A, and exon 17 D820Y deletions was measured using both ddPCR and a targeted NGS for 21 time points (assessed in [30] for patient #1 and at two time points for patient #3: d294, d599). Thus, 42 matched values were correlated. There was a significant positive correlation between the two platforms, as displayed by Spearman‘s correlation. In general, MAFs detected with ddPCR were higher than those detected with NGS. At ten time points, ctDNA remained undetected by NGS in contrast to ddPCR (empty circles). (**B**) Enlarged view of (**A**), showing multiple ctDNA values of low MAFs detected by ddPCR, which remained negative in NGS (empty circles).

We next correlated mutant allele frequencies (MAF) obtained by NGS with those obtained by ddPCR for matched time points using data from the present study and the previous [30] (Supplementary Materials Table S3). The NGS MAF correlated well with the results from ddPCR (Figure 5A,B). Particularly, we examined matched samples for two mutations in patient #1 (exon 11 deletion and exon 13 V654A) and three mutations in patient #3 (exon 11 deletion, exon 13 V654A, and exon 17 D820Y) using ddPCR and NGS (Supplementary Materials Table S3, Figure 4E). Comparison of NGS and ddPCR results showed a highly significant correlation (ρ = 0.6047; *p* = 0.0036; Figure 5A,B). MAF values of ctDNA assessed with NGS were generally lower than values assessed with ddPCR (mean 1.07% [SD 2.13] for ddPCR vs. mean 0.54% [SD 1.68] for NGS; Supplementary Materials Table S3). For 16/21 data time points, MUT ctDNA was detectable with ddPCR, whereas NGS was able to detect a MUT signal in only 6/21 data time points (Figure 4A,E and Figure 5A,B; Supplementary Materials Table S3).

## 3. Discussion

Therapy for GISTs depends on tumor stage, location, size, and mitotic count [37]. Localized tumors are surgically resected, while those with imatinib-responsive cKIT mutations and high risk of relapse are then also treated with adjuvant imatinib [38,39,40]. Locally advanced tumors for which tissue sequencing reveals imatinib-responsive cKIT mutations are treated with neoadjuvant imatinib. Metastatic disease requires treatment with imatinib [37,41]. Although the major proportion of patients with unresectable or metastatic GISTs initially responds to imatinib, progression-free survival (PFS) is limited to 18–24 months [41,42,43,44,45].

Testing for cKIT/PDGFRA mutational status is essential as it predicts patient responses to imatinib [46]. Whenever possible, patients should receive a fresh tumor biopsy at the time of progression. In 50–80% of cases (or more), imatinib resistance is mediated by additional cKIT or PDGFRA mutations [2,7,35,36,47], which might pre-exist and become selected with treatment or else might arise de-novo during treatment [12]. A mutation-based algorithm predicts therapy response and strongly impacts decision-making. In case of secondary mutations in cKIT exon 13/14, sunitinib shows activity, whereas patients harboring cKIT exon 17/18 mutations might benefit from regorafenib [13,14]. Notably, there is currently no single substance proved to be capable of inhibiting all known cKIT mutations [9], and only avapritinib induces responses in GIST patients with PDGFRA D842V. Thus, changes in genetic composition during treatment determine the response to second- and third-line treatment, and genetic testing is mandatory to direct initial and subsequent lines of treatment. 

Surgical biopsies are still considered the gold standard for cancer diagnosis and treatment [22]. Yet, tumor tissue sampling only delivers a static and spatially limited representation, not fully echoing the intra- and intertumoral genetic heterogeneity that is characteristic of advanced malignancies [22]. We have previously shown that specific detection of pre-identified ctDNA mutations via L-PCR and ddPCR in patients with localized, advanced, or metastatic GISTs is feasible [30,48]. Particularly, the combination of L-PCR and ddPCR improved the ctDNA detection rate by 92%, with ddPCR demonstrating superior sensitivity and specificity [30]. In the latter study, we focused on primary cKIT and PDGFRA mutations including the highly heterogenous cKIT exon 11 mutations. For these, we conducted a drop-off assay to determine sensitivity to imatinib and allow treatment monitoring [30]. In the current study, we complemented our previous ddPCR assay panel by adding an assay to detect cKIT exon 13 K642E and by redesigning the cKIT exon 9 A502-Y503 detection assay to be a more practical WT/MUT probe-based assay; these two mutations constitute the most frequent imatinib-sensitive cKIT exon 13 and 9 mutations, respectively, in GISTs [3,5,7]. Furthermore, we designed the cKIT exon 13 V654A and T670I, as well as the cKIT 17 drop-off assays to enable detection of mutations mediating imatinib resistance. Notably, approximately two-thirds of patients with acquired imatinib resistance harbor additional cKIT mutations, as assessed by sequencing from tissue specimens [49]. Thus, our new imatinib resistance assays cover >90% of these additional mutations (cKIT exon 13 V654A, exon 14 T670I, and the multiple exon 17 mutations covered by the exon 17 drop-off assay). 

The proportion of ctDNA contributing to cfDNA can be substantially low. A general prerequisite for ctDNA-based assays is therefore the detection of very low-frequency ctDNA amongst total cfDNA. For four out of five of the newly designed assays, the LoD ranged from 0.1 to 0.005%, producing minimal ratios of 1:1009 to 1:17,322. The LoD ratio for the cKIT exon 17 drop-off assay was 1:610, which still corresponds to a minimal ratio of <0.2%. The amount of WT DNA fragments from healthy controls was between 50 and 600 fragments/µL. The intended minimal dilution of 1:10,000 (1 MUT strand in 10,000 WT strands) allows for the detection of minimal amounts of MUT DNA in four-digit WT DNA, which would allow for the detection of single fragments of MUT DNA in a sample. However, the actual LoD is still set by the limited amount of plasma available for analysis. 

In our study, as per study protocol, we extracted 18 mL of peripheral blood from each patient, which might comprise a rather large amount of blood in a routine clinical setting. However, in our study, this was necessary to provide sufficient material for ddPCR measurement of primary and secondary mutations, NGS, and back up samples. Notably, for each 1–2 mL of plasma, at least 60 µL of DNA eluate were obtained—of which 7 µL were sufficient per ddPCR assay run (i.e., the DNA derived from approximately 100–200 µL of plasma/assay run). For NGS analysis, 60 µL of DNA eluate were subsequently used for NGS library generation, corresponding to 1–2 mL of plasma. In other words, less material would be required for ddPCR and NGS in a routine clinical setting than was defined in our study protocol.

Both PCR-based techniques and NGS were shown to exhibit high analytical sensitivity [23,24,30,33,48,50]. Particularly, BEAMing and ddPCR are characterized by sensitivities reaching levels between 0.01 and 0.1% [30,33,48]. Technical developments within the last few years have raised the sensitivity of NGS to similar levels [23,24,50,51]. NGS harbors a range of advantages compared to ddPCR, in particular the unbiased detection of multiple ctDNA mutations in parallel. Still, NGS is more expensive and time-consuming, and it requires extensive bioinformatics compared to ddPCR. In addition, as shown recently, NGS panel (TST26) testing of GIST specimens either missed or inaccurately called complex insertion/deletion variants in cKIT exon 11 that were accurately identified by non-NGS methods [52]. Varying rates of ctDNA detection, defined by detection at at least one time point for each patient, were reported for NGS in GISTs [11,24,27,28,29,50,51]: detection rates between 0.0 and 24% were reported for localized disease or smaller tumors (<10 cm) [24,27,51], with a range of 38.5–100% for larger tumors (>10 cm) or metastatic disease [11,25,28,30,50]. Thus, the detection rates of ctDNA by NGS were higher for patients with metastatic disease, but showed high variability. For ddPCR, detections rates reported for localized disease were between 0% and 75% [30,51,53]. The detection rate by ddPCR in metastatic GISTs ranged between 69.2 and 92.9% [30,50,51,53]. The American Society of Clinical Oncology recently recommended performing cross-platform validation of ctDNA detection [54]. Notably, within the above-mentioned studies only Serrano et al. performed a cross-validation of ctDNA results obtained by NGS and ddPCR [50,51]. In one study, the detection rate of ctDNA by ddPCR was identical to NGS (90%) [50]. However, median MAFs in ctDNA-positive patients were higher from ddPCR than from NGS (5.3% vs. 1.3%) [50]. In the second study, the detection rate was higher with ddPCR than with NGS (69.2% vs. 38.5%) [51]. 

In this study, the dynamics of ctDNA fragment numbers and MAFs of cKIT mutations fitted very well to the clinical course. Specifically, in the first patient, an increase in V654A levels accompanied radiological progression. In the second patient, a cKIT exon 17 Y823D mutation from a liver specimen after progression upon sunitinib administration was detected in plasma samples, with a lead time of 26 months. The previously obtained results from NGS [30] generally correlated well with the ddPCR results generated in the present study, with a correlation coefficient of 0.6057. Notably, in matched time points we observed a substantially higher overall detection rate for ddPCR (76%; 16/21 time points) compared to NGS (29%; 6/21 time points; Supplementary Materials Table S3). As for the study by Serrano et al. [50], mean and median MAFs assessed by NGS were lower than those assessed by ddPCR in paired measurements. Thus, the higher rate of positively detected data time points by ddPCR, for both primary and additional cKIT mutations at similar amounts of cfDNA input, underlines the power of ddPCR with regard to highly sensitive ctDNA detection. On the other hand, advantages of NGS compared to ddPCR include its ability to detect genome-wide mutations and clonal heterogeneity in a single assay [23]. Therefore, there is great potential for integrating ddPCR and NGS into a combined biomarker strategy for GISTs, thus combining the relative advantages of each platform. NGS represents the diagnostic gold standard in tissue genotyping and might be used for ctDNA genotyping in previously treated patients at progression, to assess the mutational landscape and the emergence of resistance mutations and thus guide further therapy. Digital droplet PCR might be used for the robust and sensitive detection of previously known single or multiple mutations, to monitor treatment response and detect stereotypic mutations that mediate imatinib resistance. Our panel of cKIT exon 13 V654, exon 14 T670I and exon 17 drop-off assays cover >90% of mutations that emerge with resistance to imatinib. 

Limitations of the current study are its retrospective character and, particularly, the small sample size. A larger and more-homogenous patient cohort with access to enough plasma material is required for head-to-head comparisons of the two platforms. The value of early detection of ctDNA recurrence before radiologic relapse, or of the emergence of novel specific mutations, has to be determined in a larger cohort within a prospective trial. For this purpose, we will apply our assays within a currently ongoing, prospective, multicenter trial that evaluates the significance of ctDNA detection for response monitoring and relapse prediction (German clinical trial registry No. DRKS00023192), which will include 100 patients. 

Together, we provide a complemented set of ddPCR assays that can be used in a clinical setting to monitor treatment response and detect stereotypic mutations mediating treatment resistance in GISTs. We show that ddPCR and NGS faithfully detect secondary cKIT mutations with correlating MAF values, with both methods reflecting clinical course and dynamic changes preceding clinical progression. In the future, both techniques might facilitate disease monitoring and dynamic treatment stratification in GISTs; NGS can be used for ctDNA genotyping and ddPCR can be used to monitor pre-identified mutations and stereotypic resistance mutations.

## 4. Materials and Methods

### 4.1. Digital Droplet PCR

ddPCR assays were created in accordance with the Digital MIQE Guidelines dictating the minimal requirements for publishing quantitative digital PCR experiments [55]. Customized primer pairs and locked nucleic acid (LNA) or standard probes for wildtype (WT) or mutation (MUT), respectively, were created in Beacon Designer v.8.20 software (Premier Biosoft, Palo Alto, CA, USA) to detect substitutions, deletions, insertions, or duplication mutations typically present in GIST patients (Supplementary Materials Table S1). Human genomic DNA (Roche Diagnostics, Mannheim, Germany) served as the WT background and double-stranded recombinant DNA fragments (gBlocks, IDT, Coralville, IA, USA) were used for MUT-specific, positive controls (Supplementary Materials Table S1). Primer pairs and 6-carboxyfluorescein (FAM)- or hexafluorescein (HEX)-labeled LNA/standard probes were purchased from IDT DNA Technologies (Supplementary Materials Table S1). Temperature gradient PCR was conducted to determine optimal conditions for each mutation assay (Table 1). In each ddPCR reaction, 11 µL of Supermix for Probes (No dUTP) (Bio-Rad Laboratories GmbH, Munich, Germany) was added to 0.22 µL of primers (forward and reverse, 900 nM final concentration each), 1.1 µL of probes (WT and MUT, or reference and deletion, respectively; 250 nM final concentration each), and template DNA. Molecular-biology-grade H_2_O was added to reach a total volume of 22 µL. Twenty µL were used for each well. The droplets were generated in the Automated Droplet Generator (QX200TM AutoDG, Bio-Rad Laboratories GmbH). Subsequently, the generated droplets were thermally cycled (C1000 TouchTM Thermal Cycler, Bio-Rad) as follows: 1. 10 min at 95 °C; 2. 40–50 cycles of 30 s at 95 °C followed by 60–90 s at the predetermined optimal T (°C); and 3. 10 min at 98 °C (Table 1). Finally, the fluorescence signal of each droplet was measured using the QX200TM Droplet Reader (Bio-Rad Laboratories GmbH). Data were analyzed with the QuantaSoftTM software Analysis Pro (Bio-Rad Laboratories GmbH).

### 4.2. Assay Validation

Assay sensitivity represents an assay’s ability to detect MUT DNA in a large amount of WT background DNA. To determine the limit of detection (LoD), dilution steps were performed. To this end, 66.6 ng of WT human genomic DNA (Roche Diagnostics) (corresponding to approximately 20,000 copies of the WT gene) was used. MUT DNA molecules (gBlocks, IDT) carrying 2 to 1000 copies of the target mutation were spiked in to obtain decreasing dilutions of the target sequence (1:20 to 1:10,000, respectively) (Supplementary Materials Table S2.). Within this dilution series, the minimal measured ratio that proved statistically significant in an unpaired, two-tailed *t*-test compared to no-template control (NTC) or WT-only control (background) was defined as the LoD [56]. In all experiments, wells containing NTC, WT-only controls, and a mix of WT and MUT templates (with fixed template amounts) were run concurrently to test for background and non-specific binding, i.e., false-positive reactions. To determine the limit of blank (LoB), plasma from healthy donors (*n* = 10 per assay) was collected. From each donor, 18 mL of peripheral blood were collected in EDTA tubes, and circulating free DNA (cfDNA) was isolated from the derived plasma (Sarstedt, Nümbrecht, Germany). The measured mean of MUT cfDNA fragments in ddPCR was calculated and the 3× standard deviation was added to receive the LoB: LoB = ø + 3 × δ [56,57,58].

### 4.3. Patients

From three patients with GISTs, known from tissue sequencing and active disease to have activating cKIT mutations, blood samples were measured for ctDNA. Two patients participated in the NCT01462994 trial, an open-label, nonrandomized, noninterventional, prospective, explorative, multicenter phase IIIb trial for the detection of circulating cell-free tumor DNA in the plasma of patients with active GIST-harboring activating mutations of cKIT or PDGFRA [30]. The study was approved by the responsible Institutional Review Boards (Technical University of Munich, 5108/11) and registered under Eudra-CT No. 2011-002544-27 and ClinicalTrials.gov NCT01462994. The third patient participated in the local molecular tumor board program of the University Medical Center Freiburg. This study was approved by the responsible Institutional Review Board (University of Freiburg, No. nr. 369/19). All subjects (patients and healthy donors) provided written, informed consent. The study was conducted in accordance with Good Clinical Practice and the Declaration of Helsinki. 

Tumor-specific cKIT mutations were determined from genomic DNA extracted from tumor tissue by Sanger sequencing. From each patient, 18 mL of peripheral blood were collected in EDTA tubes, and then circulating free DNA (cfDNA) was isolated from the derived plasma (Sarstedt, Nümbrecht, Germany). This volume (18 mL peripheral blood) was specified in our clinical trial protocol (ClinicalTrials.gov NCT01462994) to ensure sufficient material for cfDNA extraction, digital droplet PCR runs (including the primary mutation and secondary mutations), NGS analysis, and a backup sample. After two centrifugation steps (10 min at 800× *g*, and 1000× *g*, respectively), the obtained cell-free material was stored at −80 °C until the isolation process. For cfDNA isolation, 1–2 mL of plasma were further processed (QIAsymphony Circulating DNA KIT; QIAsymphonySP, Qiagen, Hilden, Germany), yielding at least 60 µL of DNA eluate per sample. The DNA content was determined using a Qubit fluorometer (Qubit™ dsDNA HS-Kit; Thermo Fisher Scientific, Schwerte, Germany) according to the manufacturer’s instructions. The isolated DNA was stored at −20 °C. A total of 7 µL of isolated DNA eluate were added to each well for ddPCR. Eleven plasma samples from three patients were measured for cKIT mutations by ddPCR. In two patients, ctDNA levels were additionally measured by a customized NGS panel, as previously reported [30]. For NGS, at least 60 µL of isolated DNA eluate were used for library preparation. For this purpose, cfDNA was concentrated using a SpeedVac (Thermo Fisher Scientific, Waltham, MA, USA), followed by quality control with a Fragment Analyzer (Agilent Technologies, Santa Clara, CA, USA). Separation profiles showed fragmentation with a prominent peak at 160–200 bp and nucleasomal laddering typical for cfDNA. After passing quality control, the samples were utilized for further experiments. Further procedures involving NGS and data analysis were performed as described previously [30].

### 4.4. Statistics

Measurements were taken at least in quadruplets and the mean was used for analysis. Positive droplets in the LoD assays were rated statistically significant if an unpaired, two-tailed *t*-test produced a *p* < 0.05 compared to NTC or WT. The fractional WT or MUT-strand droplet distribution was calculated using QuantaSoft software Analysis Pro and the Poisson distribution [34], compensating for the fact that more than one copy of template may have been present in some partitions [55]. Correlation between the mutant allelic fraction (MAF) measured by ddPCR and NGS was characterized using Spearman’s ρ correlation coefficient.

## Figures and Tables

**Table 1:** *cKIT* mutations: Cycling conditions, limit of detection (LoD), and limit of blank (LoB).

**cKIT**										
	Mut	Exon	Temperature	Annealing Time	Pos. Signals Neg. Controls	Mean Copies Per Partition (+Variation)	Number of Partitions (Mean)	Total Volume of Partitions Measured in µL (Mean)	Limit of Detection (LoD)	Limit of Blank (LoB) Fragments/mL
1	A502-Y503 dup	9	55 °C	60 s 50 cycles	WT: 0 MUT: 0	1.33 ± 0.5	15,652.1	15.3	1:17,322 (0.006%)	2.7
2	K642E	13	55 °C	60 s 40 cycles	WT: 0.3 MUT: 0.3	0.80 ± 0.4	18,029.6	17.1	1:1068 (0.09%)	6.70
3	V654A	13	52 °C	90 s 50 cycles + 1 µL MgCl_2_	WT: 0 MUT: 0	0.82 ± 0.6	15,280.3	15.6	1:4337 (0.02%)	3.70
4	T670I	14	55 °C	90 s 50 cycles	WT: 0 MUT: 1.5	1.35 ± 0.5	16,380.5	16.7	1:1009 (0.10%)	4.90
5	820f -> drop off	17	55 °C	90 s 50 cycles	WT: 0 MUT: 0	1.12 ± 0.4	15,493.7	16.7	1:610 (0.16%)	4.40

## Data Availability

Not applicable.

## Supplementary Materials

The following supporting information can be downloaded at: https://www.mdpi.com/article/10.3390/ijms24065411/s1.
