# Supplementary material for: Development of Highly Sensitive Digital Droplet PCR for Detection of cKIT Mutations in Circulating Free DNA That Mediate Resistance to TKI Treatment for Gastrointestinal Stromal Tumor (GIST)
Source: Int J Mol Sci. 2023 Mar 12;24(6):5411. doi: 10.3390/ijms24065411 (PMC10049191; doi:10.3390/ijms24065411)
Supplement: Supplementary file 1 [file ijms-24-05411-s001.zip › Suppl. Tbl 1+2, IJMS, Rassner et al.pdf]

| cKIT | | | | | |
|---|---|---|---|---|---|
| | **Mut** | **ex on** | **g-block sequences** | **primer (for/rev)** | **probes** |
| 1 | A502-Y503 dup | 9 | WT: gen. DNA (restriction enzyme: HIND III)<br>MUT: 4:54725897..54726178<br>AACTCATCTGGGCCACCGTTTGGAAAGCTAGTGGTTCAGAGTTCTATAGATTCTAGTGCATTCAAGCACAATGGCACGGTTGAATGTAAG<br>GCTTACAACGATGTGGGCAAGACTTCTGCCTAT**GCCTAT**TTTAACTTTGCATTTAAAGGTAACAACAAAGGTATATTTCTTTTTAATCCAATT<br>TAAGGGGATGTTTAGGCTCTGTCTACCATATCAGTCATGATTTTAAGTTCATTCCAACATTGACCATGTCATTTCTGGTAATACATGCATCA<br>CACCATACTGTCA | 5'-GGTTGAATGTAAGGCTTA-3'<br>5'-GACTGATATGGTAGACAGA-3' | WT (LNA):<br>5'-cttCtgCctAttTtaactt-3'<br>Mut. (LNA):<br>5'-ttctGccTatGccTattt-3' |
| 2 | K642E | 13 | WT: gen. DNA (restriction enzyme: HAE III)<br>MUT: 4:54727828..54728293<br>GCTTGACATCAGTTTGCCAGTTGTGCTTTTTGCTAAAATGCATGTTTCCAATTTTAGCGAGTGCCCATTTGACAGAACGGGAAGCCCTCAT<br>GTCTGAACTC**G**AAGTCCTGAGTTACCTTGGTAATCACATGAATATTGTGAATCTACTTGGAGCCTGCACCATTGGAGGTAAAGCCGTGTC<br>CAAGCTGCCTTTTATTGTCTGTCAGGTTATCAAAACATGACATTTTAATATGATTTTGGCAATGCTAGAT | 5'-GTGCCCATTTGACAGAAC-3'<br>5'-GCTCCAAGTAGATTCACAATA-3' | WT (LNA):<br>5'-tctGaaCtcAaaGtcctga-3'<br>Mut. (LNA):<br>5'-tctGaaCtcGaaGtcctg-3' |
| 3 | V654A | 13 | WT: gen. DNA (restriction enzyme: HAE III)<br>MUT: 4:54727994..54728245<br>CATGTTTCCAATTTTAGCGAGTGCCCATTTGACAGAACGGGAAGCCCTCATGTCTGAACTCAAAGTCCTGAGTTACCTTGGTAATCACATG<br>AATATTG**C**GAATCTACTTGGAGCCTGCACCATTGGAGGTAAAGCCGTGTCCAAGCTGCCTTTTATTGTCTGTCAGGTTATCAAAACATGAC<br>ATTTTAATATGATTTTGGCAATGCTAGATTATAAACTGCTTGGAAGATTTTTTTACCCAGACTGTTGTTC | 5'- CCTGAGTTACCTTGGTAA -3'<br>5'- GCAGTTTATAATCTAGCATTG -3' | WT (LNA):<br>5'-tgaAtaTtgTgaAtctact 3'<br>Mut. (LNA):<br>5'-tgaAtaTtgCgaAtctact -3' |
| 4 | T670I | 14 | WT: gen. DNA (restriction enzyme: HIND III)<br>MUT: 4:54729128..54729502<br>GACCACCCTTGGGTATTTTTATGGGAGGCAGAATTAATCTATATATCTCACCTTCTTTCTAACCTTTTCTTATGTGCTTTTAGGGCCCACCC<br>TGGTCATTA**T**AGAATATTGTTGCTATGGTGATCTTTTGAATTTTTTGAGAAGAAAACGTGATTCATTTATTTGTTCAAAGCAGGAAGATCATG<br>CAGAAGCTGCACTTTATAAGAATCTTCTGCATTCAAAGGAGTCTTCCTGGTAAGACTGATTTACAT | 5'- CCTTCTTTCTAACCTTTTCTTA -3'<br>5'- CTTCCTGCTTTGAACAAA -3' | WT (LNA):<br>5'- cctggTcaTtaCagAatat -3'<br>Mut. (anti-sense; LNA):<br>5'- ctggTcaTtaTagAatatt -3' |
| 5 | 820 drop off here: N822K-A | 17 | WT: gen. DNA (restriction enzyme: HIND III)<br>MUT: 4 4:54733008..54733321<br>TTTTCACTCTTTACAAGTTAAAATGAATTTAAATGGTTTTCTTTTCTCCTCCAACCTAATAGTGTATTCACAGAGACTTGGCAGCCAGAAATA<br>TCCTCCTTACTCATGGTCGGATCACAAAGATTTGTGATTTTGGTCTAGCCAGAGACATCAAGAATGATTCTAA**A**TATGTGGTTAAAGGAAA<br>CGTGAGTACCCATTCTCTGCTTGACAGTCCTGCAAAGGATTTTTAGTTTCAACTTTCGATAAAAATTGTTTCCTGTGATTTTCATAATGTAAA<br>TCCTGTCTAGGGATATCACACATTTTAGCAGTCAAAT | 5'- GCAGCCAGAAATATCCTC -3'<br>5'- TAGACAGGATTTACATTATGAA -3' | Ref. (LNA):<br>5'-ttaCtcAtgGtcGgatca -3'<br>WT-specific (LNA):<br>5'-tgaTtcTaaTtaTgtggtt -3' |

**Supplementary Table S1. gBlock, primer and probe sequences for ddPCR assays of cKIT mutations**

| Percentage | MUT cps/well | WT cps/well |
|---|---|---|
| **5** | 1000 | 20.000 |
| **2.5** | 500 | 20.000 |
| **1** | 200 | 20.000 |
| **0.5** | 100 | 20.000 |
| **0.25** | 50 | 20.000 |
| **0.125** | 25 | 20.000 |
| **0.0975** | 19.5 | 20.000 |
| **0.05** | 10 | 20.000 |
| **0.01** | 2 | 20.000 |

**Supplementary Table S2. Dilution for determination of the limit of detection (LoD)**
